# Sodium–glucose cotransporter 2 inhibition suppresses HIF-1α-mediated metabolic switch from lipid oxidation to glycolysis in kidney tubule cells of diabetic mice

**DOI:** 10.1038/s41419-020-2544-7

**Published:** 2020-05-22

**Authors:** Ting Cai, Qingqing Ke, Yi Fang, Ping Wen, Hanzhi Chen, Qi Yuan, Jing Luo, Yu Zhang, Qi Sun, Yunhui Lv, Ke Zen, Lei Jiang, Yang Zhou, Junwei Yang

**Affiliations:** 1 Center for Kidney Disease, Second Affiliated Hospital, Nanjing Medical University, Nanjing, China; 2 State Key Laboratory of Pharmaceutical Biotechnology, Nanjing University Advanced Institute of Life Sciences, Nanjing, China

**Keywords:** End-stage renal disease, Diabetes complications

## Abstract

Inhibition of sodium–glucose cotransporter 2 (SGLT2) in the proximal tubule of the kidney has emerged as an effective antihyperglycemic treatment. The potential protective role of SGLT2 inhibition on diabetic kidney disease (DKD) and underlying mechanism, however, remains unknown. In this study, metabolic switch was examined using kidney samples from human with diabetes and streptozocin (STZ)-induced experimental mouse model of diabetes treated with or without SGLT2 inhibitor dapagliflozin. Results were further validated using primarily cultured proximal tubule epithelial cells. We found that DKD development and progression to renal fibrosis entailed profound changes in proximal tubule metabolism, characterized by a switch from fatty acid utilization to glycolysis and lipid accumulation, which is associated with the increased expression of HIF-1*α*. Diabetes-induced tubulointerstitial damage, such as macrophage infiltration and fibrosis, was significantly improved by dapagliflozin. Consistent with the effects of these beneficial interventions, the metabolic disorder was almost completely eliminated by dapagliflozin. The increased level of HIF-1α in renal proximal tubule was nearly nullified by dapagliflozin. Moreover, dapagliflozin protects against glucose-induced metabolic shift in PTCs via inhibiting HIF-1α. It suggests that SGLT2 inhibition is efficient in rectifying the metabolic disorder and may be a novel prevention and treatment strategy for kidney tubule in DKD.

## Introduction

Diabetic kidney disease (DKD) is the primary cause of chronic kidney disease (CKD) and end-stage renal disease (ESRD)^1^. Although the incidence of DKD is stabilizing, the absolute number of individuals affected continues to increase in consistent with the global diabetes pandemic^2^ and many diabetic individuals will require replacement therapies or die prematurely from cardiovascular events^3^. DKD is a syndrome that is characterized by leakage of albumin into urine, changes in glomerular filtration rate (GFR), and glomerular sclerosis^4,5^. However, the development of tubulointerstitial fibrosis is the critical predictor of the progression of DKD to ESRD^6^.

The available clinical management of hyperglycemia, hypertension, and dyslipidaemia stabilizes DKD without improvement of kidney function, indicating a significant treatment gap^1^. Sodium–glucose cotransporter 2 (SGLT2) inhibitors are a new class of an antidiabetic drug. SGLT2 is expressed mainly in proximal convoluted tubular epithelial cells and reabsorbs of >90% of filtered glucose^7^. In the follow-on trials for evaluation of the cardiovascular safety, secondary effects of the SGLT2 inhibitors to reduce albuminuria and decline in GFR promoted further exploration into their potential usage in DKD^8–10^. SGLT2 inhibition lessens glucose-coupled sodium reabsorption, normalizes solute delivery to macula densa, restores tubuloglomerular feedback, reverses afferent arteriole vasodilation and therefore protects glomerular hemodynamics^11,12^. However, as the direct target, how the proximal tubular epithelial cell is altered by SGLT2 inhibitor and whether the alternation has therapeutic effects remain to be elucidated.

The highly metabolic kidney tubules equipped with plenty of mitochondria require large amounts of ATP for their normal task of reabsorption^13,14^. In the tubulointerstitial compartment under physiological conditions, ATP is mainly generated via oxidative phosphorylation (OXPHOS) of fatty acid (FA) and ketone, and consumes a large amount of molecular oxygen^13,15,16^. However, in diabetic conditions, the altered delivery of metabolic substrates and oxygen to the kidney results in a shift to glucose oxidation^17,18^ and hypoxia^19^. Hypoxia-inducible factor (HIF) plays a key role coordinating the adaptive metabolic reprogram from mitochondrial OXPHOS to glycolysis under pathological condition of decreased oxygen availability^20,21^. HIF-1α is ubiquitously expressed and regulated by oxygen-dependent posttranslational hydroxylation by prolyl hydroxylase domain-containing (PHD-containing) enzymes. HIF-1α is stabilized as oxygen concentration declines and regulates expression of target genes, including glycolytic enzymes and pyruvate dehydrogenase (PDH) kinase (PDK)^22^. HIF-1α stabilization inhibits peroxisome proliferator-activated receptor α (PPARα)^23^ and acyl-CoA dehydrogenases^24^ and leads to reduced fatty acid oxidation (FAO). These observations support a central role for HIF in regulation of metabolism in the kidney tubule.

In this study, we identified a novel mechanism by which SGLT2 inhibition is efficient in renal protection by normalizing the metabolic gene program dependent on HIF-1α. By this mechanism, SGLT2 inhibition might reduce injury in the tubulointerstitium and mitigate progression of DKD to ESRD.

## Materials and methods

### Animals

All animal experimentation was conducted in accordance with the guidelines of the institutional Animal Care and Use Committee of the National Institutes of Health at Nanjing Medical University. Male CD-1 mice aged 6–8 weeks were purchased from Viral River Laboratory (Beijing, China). Animals were housed in the animal facilities in Nanjing Medical University with a 12 h: 12 h light–dark cycle and free access to food and water. A diabetic status was induced by intraperitoneal injection of 40 mg/kg of streptozotocin (STZ, S0130, Sigma Aldrich, St. Louis, MO, USA) for 3 consecutive days. Fasted blood glucose level was determined 2 weeks later, and diabetic status was established by the manifestation of polyuria, weight loss, and fasted blood glucose level greater than 16.7 mmol/l. The diabetic mice were randomly assigned into four groups: diabetic mice treated with vehicle, losartan (10 mg kg^−1^ d^−1^, HY-17512A, MCE, Monmouth Junction, NJ, USA), dapagliflozin (1 mg kg^−1^ d^−1^, PharmaBlock Science, Nanjing, China), and dapagliflozin (5 mg kg^−1^ d^−1^). Losartan and dapagliflozin were administrated by oral gavage. Mice were killed after 12 weeks of treatment. Serum and urine were collected, and kidneys were harvested for further analysis. No blinding was done during the treatment.

### Human subjects

The human study protocol conformed to the ethical guidelines of the 1975 Declaration of Helsinki as reflected in a priori approval by the Ethics Committees of Nanjing Medical University for Medical Experiments. Written informed consent was obtained from every enrolled individual. Renal biopsy samples and urine samples were collected from individuals of non-diabetes and diabetes from Center for Kidney Disease of Second Affiliated Hospital of Nanjing Medical University. Individuals were clinically diagnosed with or without diabetes according to the World Health Organization criteria and further confirmed with or without DKD by pathologic evaluation of their kidney biopsy samples. Characterization of the enrolled individuals is shown in Supplementary Table S1.

### Cell culture and treatment

Primary tubular epithelial cells (PTCs) were cultured under sterile conditions from collagenase-digested cortical fragments of kidneys isolated from mice by a modification of previously described methods^25,26^. PTCs were cultured in DMEM (no glucose, 11966, Gibco, Grand Island, NY, USA) and F-12 (11765, Gibco) medium (1:1) containing 5 μg/mL of insulin, 2.75 μg/mL of transferrin, 3.35 ng/mL of selenium (41400, Invitrogen, Grand Island, NY, USA), 40 ng/mL of hydrocortisone (614157, Sigma Aldrich), 10 ng/mL of recombinant mouse epidermal growth factor (2028-EG-200, R&D Systems, Minneapolis, MN, USA), and 1% vol./vol. antibiotic solution containing 10,000 U/mL of penicillin and 0.1 mg/mL of streptomycin (15140, Sigma Aldrich). The glucose concentration in cell culture medium was 5.5 mmol/l.

PTCs were seeded on six-well culture plates to 60–70% confluence in complete medium for 16 h, and changed to serum-free medium. Cells were then exposed to the 30 mmol/l of D-glucose (G8270, Sigma Aldrich). Because of the osmotic pressure of high glucose, control cells were treated with D-mannitol (M4125, Sigma Aldrich). The glucose decline in medium is the difference between the initial and terminal glucose concentrations in the cell culture medium. Molidustat (10 μmol/l, S8138, Selleckchem, Houston, TX, USA) or dapagliflozin (2 μmol/l, PharmaBlock Science) treatment was given for the indicated time periods. Dapagliflozin was given 30 min before glucose. PTCs were transiently transfected with negative control siRNA or HIF-1α siRNA (Ibsbio, Shanghai, China) using Lipofectamine RNAiMAX transfection reagent (13778, Invitrogen) according to the manufacturer’s instructions. After transfection for 24 h, PTCs were then exposed to the high glucose. The sequences of siRNA were as follows: HIF-1α: sense 5′-GAU GGA AGC ACU AGA CAA AGU-3′; anti-sense 5′-UUU GUC UAG UGC UUC CAU CAG-3′. Negative control (N.C.): sense 5′-UUC UCC GAA CGU GUC ACG UTT-3′; anti-sense 5′-ACG UGA CAC GUU CGG AGA ATT-3′.

### Western blot analysis

Cells were lysed in 1 × SDS sample buffer. Kidney tissue was homogenized by a polytron homogenizer (Brinkmann Instruments) in RIPA lysis buffer on ice. The supernatants were collected after centrifugation at 13,000 × *g* at 4 °C for 30 min. Protein concentration was determined by bicinchoninic acid protein assay. An equal amount of protein was loaded into 10% or 15% wt/vol. SDS-PAGE, and transferred onto polyvinylidene difluoride membranes. The primary antibodies were as follows: anti-PPAR*α* (ab24509, Abcam, Cambridge, MA, USA), anti-CPT1*α* (ab128568, Abcam), anti-ACADL (ab196655, Abcam), anti-HK2 (ab76959, Abcam), anti-LDH (3558, Cell Signaling Technology, Danvers, MA, USA), anti-PDK1 (ab110025, Abcam), anti-HIF-1α (ab1, Abcam), anti-PHD2 (4835, Cell Signaling Technology), and anti-tubulin (T6074, Sigma Aldrich). Western blot was performed at least three times independently. Chemiluminescence is applied for detecting proteins on western blot membranes. The enhanced chemiluminescent ECL substrate (32209, Thermofisher Scientific, Carlsbad, CA, USA) enables immunodetection of horseradish peroxidase (HRP)-conjugated secondary antibodies using an imaging system. Quantification was performed by measurement of the intensity of the signals with the aid of Image J software package.

### Real-time quantitative-PCR analysis

Quantitative polymerase chain reaction (Q-PCR) was performed using an Applied Biosystems 7300 Sequence Detection system. The total RNA of tissues was prepared using a TRIzol isolation system according to the instructions by the manufacturer (Invitrogen). The first strand of cDNA and subsequent real-time quantification were performed according to the instructions by the manufacturer (Thermofisher Scientific). All reactions were run in triplicate. The CT data were determined using default threshold settings, and the mean CT was calculated from the triplicate PCRs. The ratio of mRNA was calculated by using the equation 2^−ΔCT^, in which ΔCT = CT_treatment_–CT_control_. Sequences of primer pairs are shown in Supplementary Table S2.

### Lactate and glucose measurement

Lactate concentration of urine, kidney tissue and cell supernatant were measured using Lactate Colorimetric Assay Kit (K607-100, Biovision, Milpitas, CA, USA) according to the manufacturer’s instructions. According to the manufacturer, the detection range of lactate with this kit was above 0.04 nmol/μl. Urinary lactate level was normalized with the urine creatinine level. Glucose concentration in cell supernatant or mouse urine was measured using Glucose Colorimetric Assay Kit (K606-100; Biovision).

### Urine analysis

Urinary creatinine was determined by using a QuantiChrom creatinine assay kit according to the protocol (DICT-500; BioAssay Systems, Hayward, CA, USA). Quantikine Elisa kits were used for measurement of urinary albumin (E90-1134, Bethyl, Montgomery, TX, USA) and urinary Neutrophil gelatinase-associated lipocalin (NGAL) (MLCN20, R&D Systems) according to the manufacturer’s instructions.

### Triacylglycerol measurement

Kidney tissue minced into small pieces was homogenized in NP40 Assay Reagent, and triacylglycerol (TG) was measured using quantification kits (10010303, Cayman Chemical, Ann Arbor, MI, USA) according to the manufacturer’s instructions.

### Histological analysis

Neutraformalin (10% vol./vol.)-fixed kidney samples were kept at 4 °C overnight. The samples were then paraffin-embedded and sectioned at 3 μm in thickness for hematoxylin and eosin (H&E), periodic Acid Schiff (PAS), Masson and Sirius Red staining. Slides were viewed with a Nikon Eclipse 80i microscope equipped with a digital camera (DS-Ri1, Nikon, Shanghai, China). For determination of glomerular tuft area and fractional mesangial area (FMA, %), at least ten randomly chosen fields under the microscope were evaluated for each mouse with Image J software, and an average score was calculated.

### Immunohistochemistry staining

Paraffin-embedded kidney sections were deparaffinized, hydrated, antigen retrieved, and endogenous peroxidase activity was quenched by 3% vol./vol. H_2_O_2_. Sections were then blocked with 10% vol./vol. normal donkey serum, followed by incubation with anti-PPAR*α* (ab24509, Abcam), anti-CPT1α (ab128568, Abcam), anti-HIF-1α (NB100-123, Novus, New York, NY, USA), anti-HK2 (ab76959, Abcam), or anti-phospho-LDH (8176S, Cell Signaling Technology) overnight at 4 °C. After wash, sections were incubation with secondary antibody for 1 h, followed by incubation with avidin–biotin complex reagents for 1 h at room temperature before being subjected to substrate 3-amino-9-ethylcarbazole (Vector Laboratories, Burlingame, CA, USA). The percentage of positive cells to the selected field was analyzed using Image Pro Plus 6.0 software. An average percentage for each section was calculated. At least ten randomly chosen fields under the microscope were evaluated for each sample, and an average score was calculated.

### Immunofluorescent staining

Cells cultured on coverslips were washed twice with cold PBS and fixed with cold methanol/acetone (1:1) for 10 min at −20 °C. After extensive washings with PBS, the cells were blocked with 0.1% vol./vol. Triton X-100 and 2% vol./vol. normal donkey serum in PBS buffer for 40 min at room temperature and then incubated with anti-HIF-1α antibodies (NB100-123, Novus), followed by staining with FITC-conjugated secondary antibodies. Cells were double stained with DAPI to visualize the nuclei.

### Lipid droplets staining

Lipid droplets were viewed by BODIPY or oil red O staining. Briefly, freshly prepared kidney tissues were OCT-embedded and sectioned at 3 μm for BODIPY (D3922, Thermofisher Scientific) staining, which was diluted in DMSO at a concentration of 1 mg/ml according to the manufacturer’s instructions. After stained with BODIPY, slides were immunostained with laminin (ab11575, Abcam) and DAPI. Oil Red O was performed according to the manufacturer’s protocol (O0625, Sigma Aldrich) on 9-μm thickness sections of fixed or frozen kidney tissue and cells cultured on coverslips. The kidney sections and cells were rinsed in distilled water and isopropanol, and stained for 15 min in the Oil Red O working solution at room temperature, then rinsed again for 1 min in 60% vol./vol. isopropanol and returned to distilled water. The slides were counterstained with hematoxylin for 1 min. The percentage of positive area to the selected field was analyzed using Image Pro Plus 6.0 software. An average positive area for each section was calculated. At least ten randomly chosen fields under the microscope were evaluated for each sample, and an average score was calculated.

### Quantitative determination of collagen in kidney tissue

Paraffin-embedded kidney tissues were stained with Sirius red F3BA and Food green FCF (Sigma Aldrich) overnight. After washing with PBS buffer, the dye was eluted from tissue sections with 0.1 N sodium hydroxide methanol. Absorbance at 540 and 605 nm was determined for Sirius red F3BA and Food green FCF-binding protein, respectively. This assay provides a simple, relative measurement of the ratio of collagen to total protein, which is expressed as micrograms per milligram of total protein.

### Statistical analysis

Statistical evaluation was carried out using the one-way ANOVA followed by the Tukey post test using GraphPad Prism (GraphPad 7.00 Software). A *p* < 0.05 value was considered to be significant. Error bars indicate SD.

## Results

### Metabolic signatures of human DKD samples

**Fig. 1:**
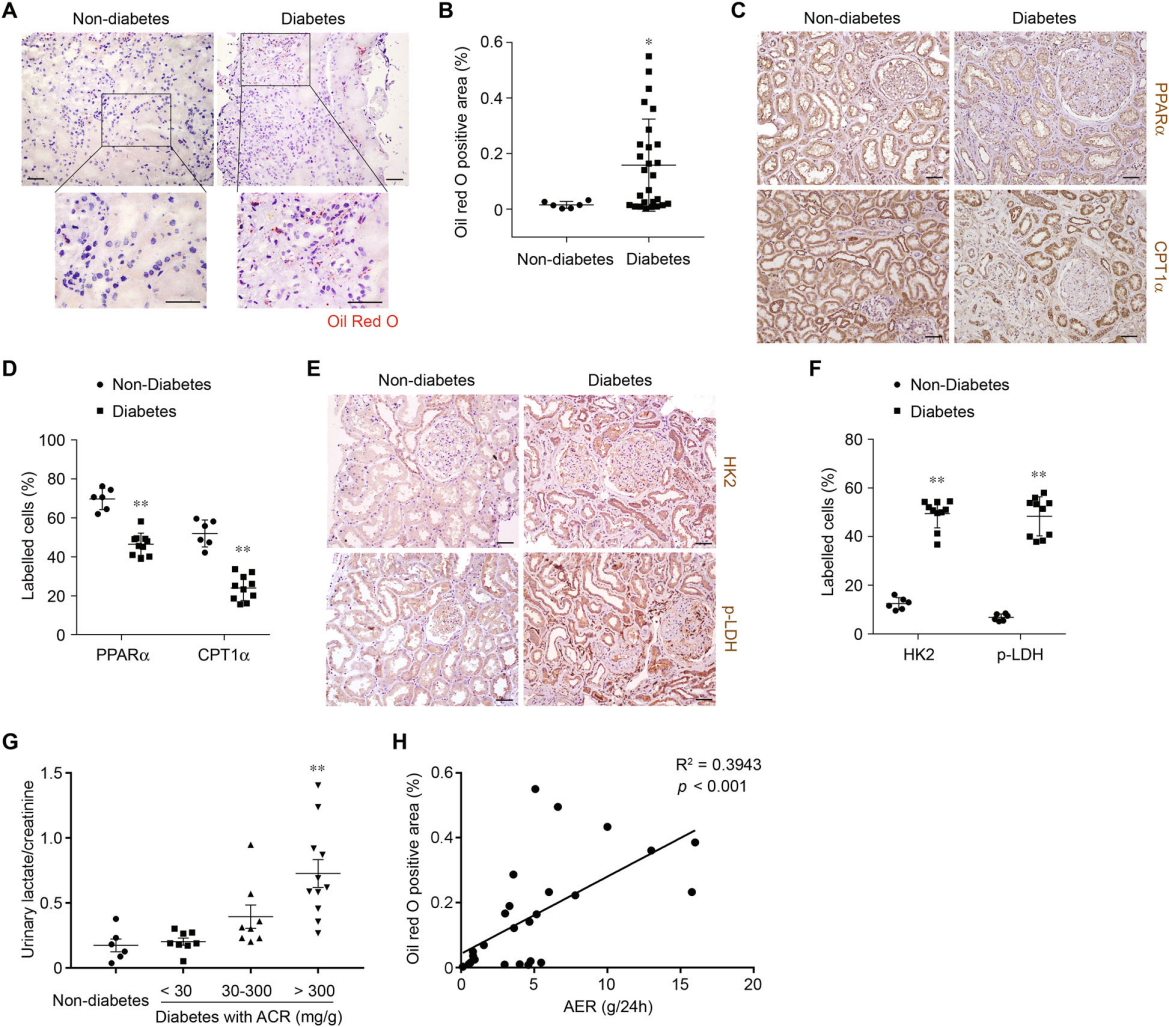
Analysis of human diabetic kidney disease identifies a metabolic switch from fatty acid oxidation to glycolysis. **a** Representative images of human kidney sections from non-diabetes and diabetes individuals with Oil Red O staining. Scale bars, 50 μm. **b** Quantification of Oil Red O-positive area in kidney samples from non-diabetes (*n* = 6) and diabetes individuals (*n* = 27). *p < 0.05 compared with non-diabetes. **c** Representative images of human kidney sections from non-diabetes and diabetes individuals with immunostaining for PPARα and CPT1α. Scale bars, 50 μm. **d** Quantification of PPARα- and CPT1α-labeled cells in kidney samples from non-diabetes (*n* = 6) and diabetes individuals (*n* = 10). **p < 0.01 compared with non-diabetes. **e** Representative images of human kidney sections from non-diabetes and diabetes individuals with immunostaining for HK2 and phospho-LDH (p-LDH). Scale bars, 50 μm. **f** Quantification of HK2- and p-LDH-labeled cells in kidney samples from non-diabetes (*n* = 6) and diabetes individuals (*n* = 10). **p < 0.01 compared with non-diabetes. **g** Quantification of lactate/creatinine ratio in urinary samples from non-diabetes and diabetes individuals with different urinary albumin/creatinine ratio (ACR). **p < 0.01 compared with non-diabetes. **h** Linear regression analysis of Oil red O-positive area versus albumin excretion rate (AER) of diabetes individuals (*n* = 27).

To identify the metabolic changes associated with DKD, we collected and analyzed microdissected human kidney samples. Characterization of the enrolled individuals was shown in Supplementary Table S1. Diabetes individuals were older, and had a significantly higher hemoglobin A1c (HbA1c) and urinary protein excretion level but lower diastolic blood pressure than non-diabetes control. We found markedly higher lipid accumulation (Fig. 1a, b) in tubulointerstitial space of diabetic individuals. A further interrogation indicated that the levels of transcriptional regulator PPARα and key enzyme carnitine palmitoyltransferase-1α (CPT1α) related to FAO were markedly lower in diabetic individuals compared with samples obtained from people without diabetes (Fig. 1c, d). Levels of key regulator of glucose utilization hexokinase (HK2) and lactate dehydrogenase (LDH) were higher in diabetic samples compared with nondiabetic controls (Fig. 1e, f). Lactate level was quite low in urine of nondiabetic individuals. However, urinary excretion level of lactate was markedly increased in diabetic individuals and was associated with albuminuria (Fig. 1g), indicating that glucose is used mainly for anaerobic glycolysis. In this set of individual samples, we used supervised correlation to analyze lipid droplets and urinary lactate with respective urinary albumin level. We found area of lipid droplet to directly correlate with albumin excretion rate (AER) (Fig. 1h). Collectively, these results identify a metabolic signature in diabetic kidneys and reveal a significant correlation between the urinary albumin and a metabolic shift from FAO to glycolysis.

### Metabolic gene switch in kidney tubule from experimental diabetes

**Fig. 2:**
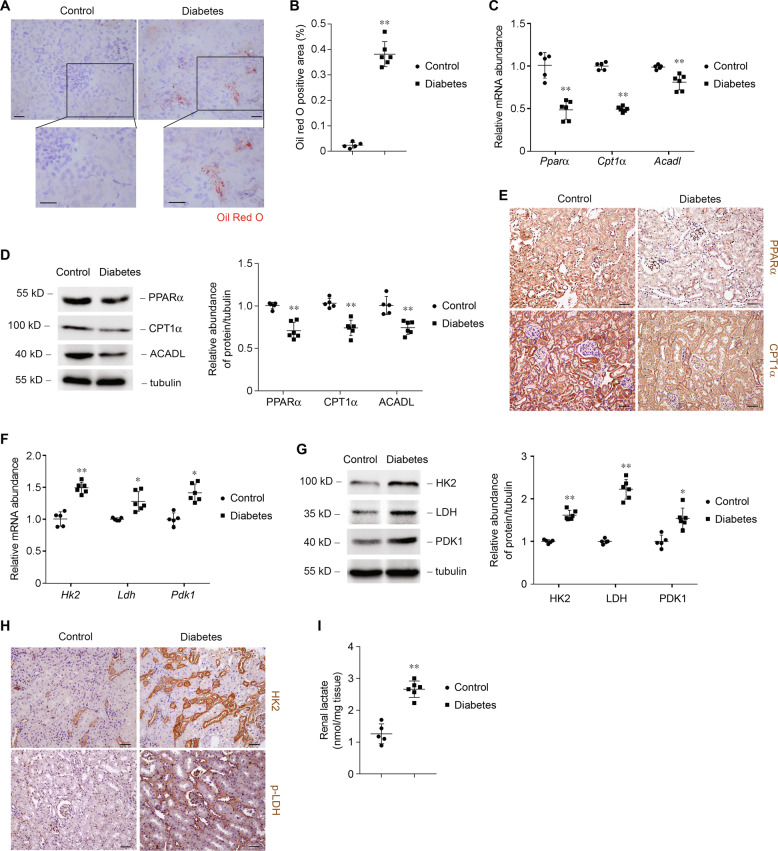
Animal model of diabetic kidney disease demonstrate a metabolic switch from fatty acid oxidation to glycolysis. **a** Representative images of mice kidney sections from control and diabetic mice with Oil Red O staining. Scale bars, 50 μm. **b** Quantification of Oil Red O-positive area in kidney samples from control (*n* = 5) and diabetic mice (*n* = 6). **p < 0.01 compared with control. **c** Relative mRNA levels of genes related to FAO (*Pparα*, *Cpt1α*, and *Acadl)* in control samples and samples from diabetic mice. ** *p* < 0.01 compared with control. **d** Western blot analysis of proteins related to FAO and relative protein expression levels in control samples and samples from diabetic mice. **p < 0.01 compared with control. **e** Representative images of mouse kidney sections from control and diabetes with immunostaining for PPARα and CPT1α. Scale bars, 50 μm. **f** Relative mRNA levels of genes related to glucose utilization in control samples and samples from diabetic mice. *p < 0.05 compared with control. **p < 0.01 compared with control. **g** Western blot analysis of proteins related to glucose utilization and relative protein expression levels in control samples and samples from diabetic mice. *p < 0.05 compared with control. **p < 0.01 compared with control. **h** Representative images of mouse kidney sections from control and diabetes with immunostaining for HK2 and p-LDH. Scale bars, 50 μm. **i** Quantification of lactate in kidney tissue samples from control and diabetic mice. **p < 0.01 compared with control.

The metabolic switch in diabetic human kidneys prompted us to analysis metabolic patterns in experimental diabetic kidney injury. For this purpose, we established streptozotocin (STZ)-induced diabetes, a well characterized mice model with progressive kidney disease. As shown in Fig. 2a, b, lipid-droplet accumulation was higher in diabetic mice compared with control mice (Fig. 2a, b). We found that transcript (Fig. 2c) and protein levels (Fig. 2d) of key FAO enzymes (PPARα, CPT1α, and ACADL) were lower in diabetic mice compared with control mice. Immune staining also revealed that the levels of PPARα and CPT1α were markedly lower in diabetic mice compared with control mice (Fig. 2e). Transcript (Fig. 2f) and protein levels (Fig. 2g, h) of key and rate-limiting enzymes of glucose utilization (HK2, LDH, and PDK1) were markedly increased in mice diabetic model compared with that in controls. The markedly higher lactate content occurred concomitantly in renal tissue from diabetic mice compared with control mice (Fig. 2i).

**Fig. 3:**
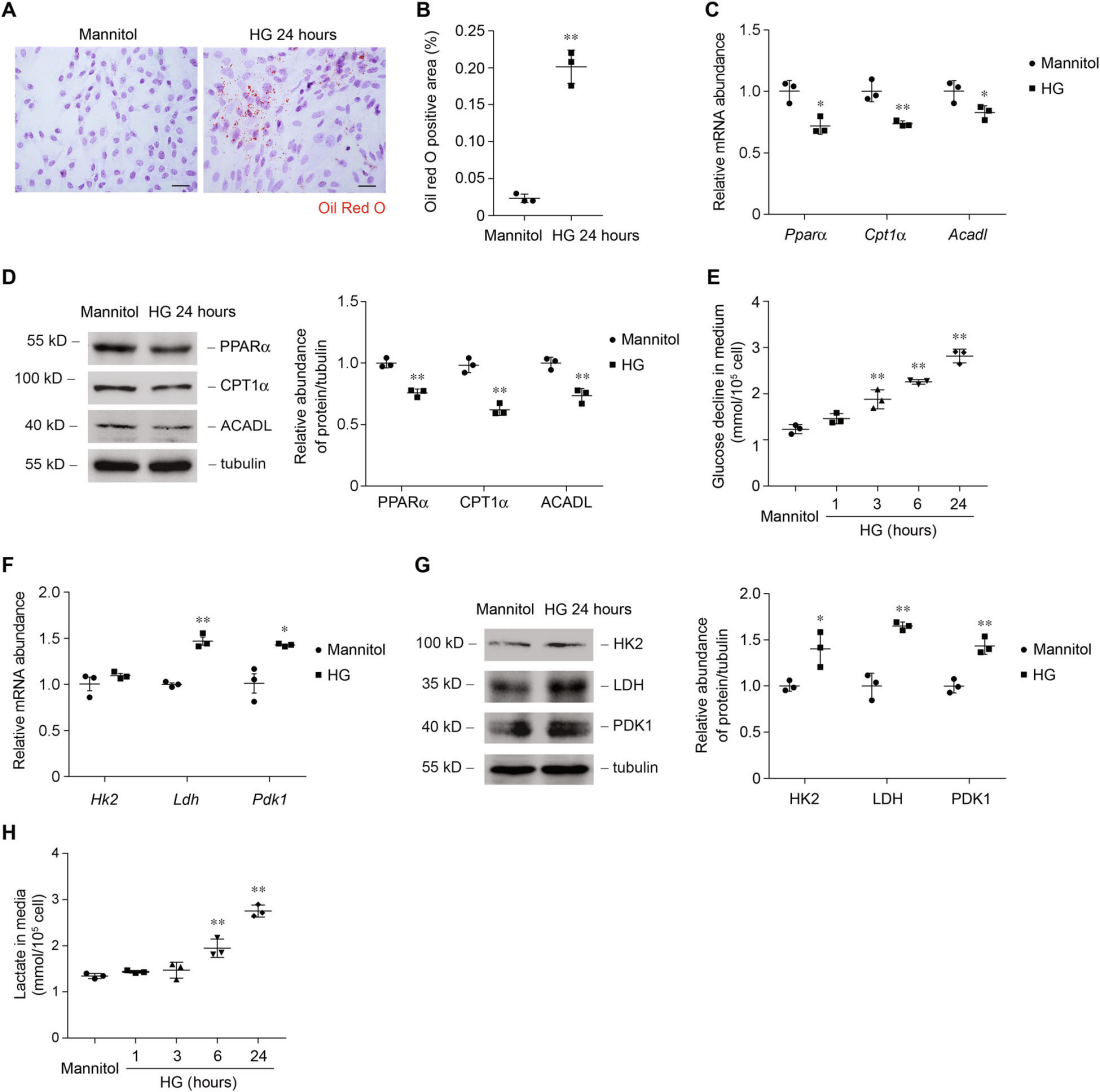
Glucose induces a metabolic switch from FAO to glycolysis in primary tubular epithelial cells (PTCs). **a** Representative images of PTCs incubated with 30 mmol/l of glucose for 24 h with Oil Red O staining. Scale bars, 25 μm. **b** Quantification of Oil Red O-positive area in PTCs incubated with 30 mmol/l of glucose for 24 h. *n* = 3 experiments. **p < 0.01 compared to control. **c** Relative mRNA levels of genes related to FAO (*Pparα*, *Cpt1α*, and *Acadl)* in PTCs incubated with 30 mmol/l of glucose for 6 h. *p < 0.05 compared with control; **p < 0.01 compared with control. **d** Western blot analysis of proteins related to FAO and relative protein expression levels in control and HG-treated PTCs. **p < 0.01 compared with control. **e** Glucose decline in medium of PTCs incubated with 30 mmol/l of glucose for various time periods, as indicated. **p < 0.01 compared with control. **f** Relative mRNA levels of genes related to glucose utilization (*Hk2*, *Ldh*, and *Pdk1*) in PTCs incubated with 30 mmol/l of glucose for 6 h. *p < 0.05 compared with control; **p < 0.01 compared with control. **g** Western blot analysis of proteins related to glucose utilization and relative protein expression levels in control and HG-treated PTCs. *p < 0.05 compared with control. **p < 0.01 compared with control. **h** Quantification of lactate in culture medium of PTCs incubated with 30 mmol/l of glucose for various time periods, as indicated. **p < 0.01 compared with control.

Next, we evaluated metabolic changes specifically in primarily cultured tubular epithelial cells (PTCs). Cells were cultured under 5.5 mmol/l (control) or 30 mmol/l (high glucose, HG) of D-glucose for 24 h. HG treatment was associated with more intracellular lipid-droplet accumulation (Fig. 3a, b). Consistently, HG treatment reduced *Pparα* mRNA (Fig. 3c) and protein (Fig. 3d) levels, as well as its downstream targets *Cpt1α* and *Acadl*. The alteration of *Pparα* and its downstream target gene expression is probably the cause of the lower FAO and accumulation of lipid in PTCs. The glucose decline in medium of PTCs was markedly increased in the condition of high glucose content (Fig. 3e). Levels of glucose metabolism-related enzymes were higher in HG-treated PTCs (Fig. 3f, g). Lactate was higher after HG treatment, indicating that glucose is used mainly for anaerobic glycolysis (Fig. 3h). We found that treatment with HG was associated with metabolic switch, phenotypes similar to what we observed in human and animal diabetic kidneys. These results indicate the characteristics of the alteration of enzymes that regulate FAO and carbohydrate metabolism, accompanied by high intracellular lipid accumulation and lactate concentration in diabetic tubule.

### Metabolic switch in diabetes is associated with increased expression of HIF-1α

**Fig. 4:**
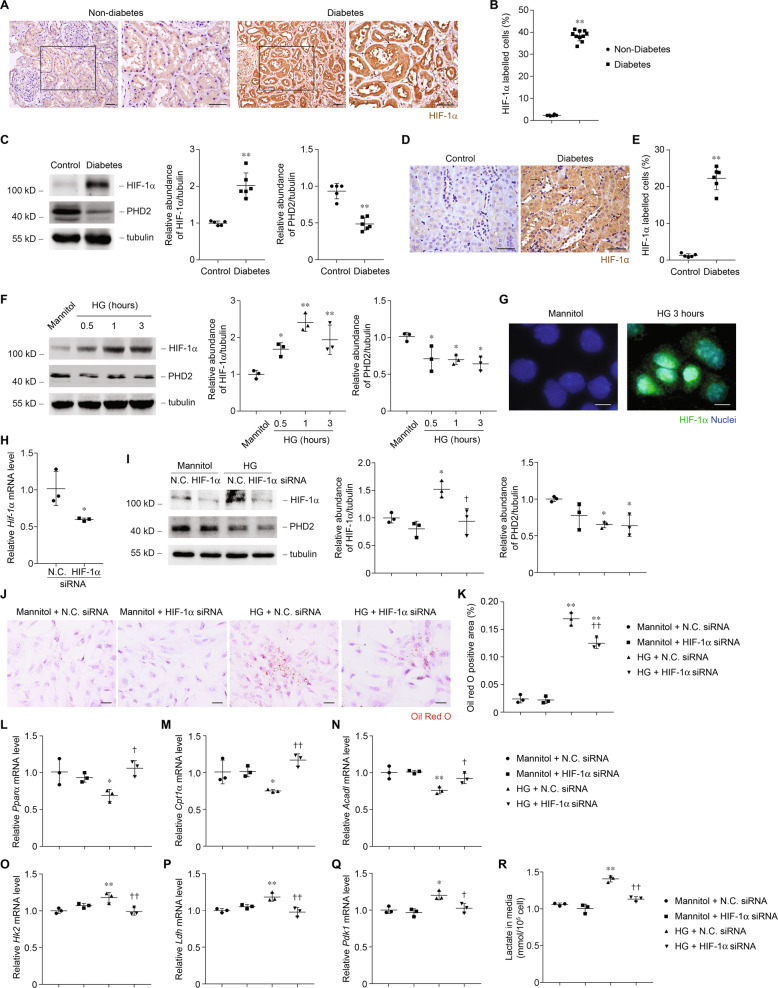
HIF-1α is a candidate transcription factor driving the transcriptional changes in diabetes. **a** Representative images of human kidney sections from non-diabetes and diabetes individuals with immunostaining for HIF-1α. Arrows indicate HIF-1α-positive nuclei. Scale bars, 50 μm. **b** Quantification of HIF-1α-lebelled cells in kidney samples from non-diabetes (*n* = 6) and diabetes individuals (*n* = 10). **p < 0.01 compared with non-diabetes. **c** Western blot analysis of HIF-1α and PHD2 protein and relative protein expression level in control samples and samples from diabetic mice. **p < 0.01 compared with control. **d** Representative images of mice kidney sections from control and diabetes with immunostaining for HIF-1α. Arrows indicate HIF-1α-positive nuclei. Scale bars, 50 μm. **e** Quantification of HIF-1α-lebelled cells in kidney samples from control (*n* = 5) and diabetic mice (*n* = 6). **p < 0.01 compared with control. **f** Western blot analysis of HIF-1α and PHD2 protein and relative protein expression level in PTCs incubated with 30 mmol/l of glucose for various time periods as indicated. *p < 0.05 compared with control. **p < 0.01 compared with control. **g** Representative images of PTCs incubated with 30 mmol/l of glucose for 3 h with immunostaining for HIF-1α. Scale bars, 10 μm. **h** Relative mRNA levels of HIF-1α in PTCs after transfection for 24 h with siRNA as indicated. *n* = 3 experiments. *p < 0.05 compared with negative control (N.C.) siRNA. **i** Western blot analysis of HIF-1α and PHD2 protein and relative protein expression level in PTCs transfected with siRNA and incubated with 30 mmol/l of glucose for 3 h. *p < 0.05 compared with control + N.C. siRNA. ^†^*p* < 0.01 compared with HG + N.C. siRNA. **j** Representative images of PTCs transfected with siRNA and incubated with 30 mmol/l of glucose for 24 h with Oil Red O staining. Scale bars, 25 μm. **k** Quantification of Oil Red O-positive area in PTCs. **p < 0.01 compared with control + N.C. siRNA. ^††^*p* < 0.01 compared with HG + N.C. siRNA. **l**–**n** Relative mRNA levels of *Pparα* (**l**), *Cpt1α* (**m**), and *Acadl* (**n**) in PTCs transfected with siRNA and incubated with 30 mmol/l of glucose for 6 h. * *p* < 0.05 compared with control + N.C. siRNA. **p < 0.01 compared with control + N.C. siRNA. ^†^*p* < 0.05 compared with HG + N.C. siRNA. ^††^*p* < 0.01 compared with HG + N.C. siRNA. **o**–**q** Relative mRNA levels of *Hk2* (**o**), *Ldh* (**p**), and *Pdk1* (**q**) in PTCs transfected with siRNA and incubated with 30 mmol/l of glucose for 6 h. *p < 0.05 compared with control + N.C. siRNA. **p < 0.01 compared with control + N.C. siRNA. ^†^*p* < 0.05 compared with HG + N.C. siRNA. ^††^*p* < 0.01 compared with HG + N.C. siRNA. **r** Quantification of lactate in culture medium of PTCs transfected with siRNA and incubated with 30 mmol/l of glucose for 24 h. **p < 0.01 compared with control + N.C. siRNA. ^††^*p* < 0.01 compared with HG + N.C. siRNA.

In mammalian cells, HIF-1α plays a key role coordinating the reprogram of metabolism from a mitochondrial oxidative to a glycolytic form. The transcriptional regulator HIF-1α, which was virtually absent in healthy kidneys, was upregulated in diseased renal tubules (Fig. 4a, b). PPARα and HK2 are well-described HIF-1α transcriptional targets, suggesting a central role for HIF-1α in the metabolic gene reprogramming of renal tubule cells. The transcript of *Hif-1α* was not changed in diabetic mice (Supplementary Fig. S1a), but the expression of HIF-1α protein was higher accompanied by the lower expression of PHD2 in kidneys from mice with diabetes (Fig. 4c). The increased expression of HIF-1α in renal tubule cells (Fig. 4d, e) preceded the change of morphology, potentially suggesting a causal role. In PTCs, similarly, expression but not transcript (Supplementary Fig. S1b) of HIF-1α is increased while PHD2 is decreased soon after HG treatment (Fig. 4f), suggesting that HIF-1α is not de novo synthesized. Immune staining shows the location of increased HIF-1α in nuclei of PTCs, indicating the transcriptional activity of HIF-1α in HG-treated PTCs (Fig. 4g).

We analyzed whether deficiency of HIF-1α could suppress metabolic switch in response to high glucose stimuli. We used siRNA against mouse *Hif-1α* to knockdown HIF-1α in wild-type PTCs (Fig. 4h). The protein expression of HIF-1α in response to high glucose was suppressed by HIF-1α siRNA transduction relative to negative control siRNA (Fig. 4i). Knockdown of HIF-1α reduced lipid accumulation (Fig. 4j, k), normalized *Pparα* repression (Fig. 4l), and FAO enzymes levels reduction (Fig. 4m–n), and reversed glycolysis enzymes levels elevation (Fig. 4o–q) and lactate production (Fig. 4r). In conclusion, the shift from FAO to glycolysis occurs in renal tubule is regulated by the activation of a HIF-1α-driven program in tubule cells.

### Dapagliflozin is renal protective in experimental diabetes

The metabolic derangement observed in kidneys from diabetic individual and animal samples prompted us to examine whether lowing the glucose level in tubular epithelial cells by SGLT2 inhibitor dapagliflozin may prohibit diabetic kidney injury. Because SGLT inhibition protects glomerular hemodynamics, we study the effects of dapagliflozin in comparison with losartan and vehicle treatment in STZ-induced diabetic mice. These interventions were applied for 12 weeks until killing.

**Fig. 5:**
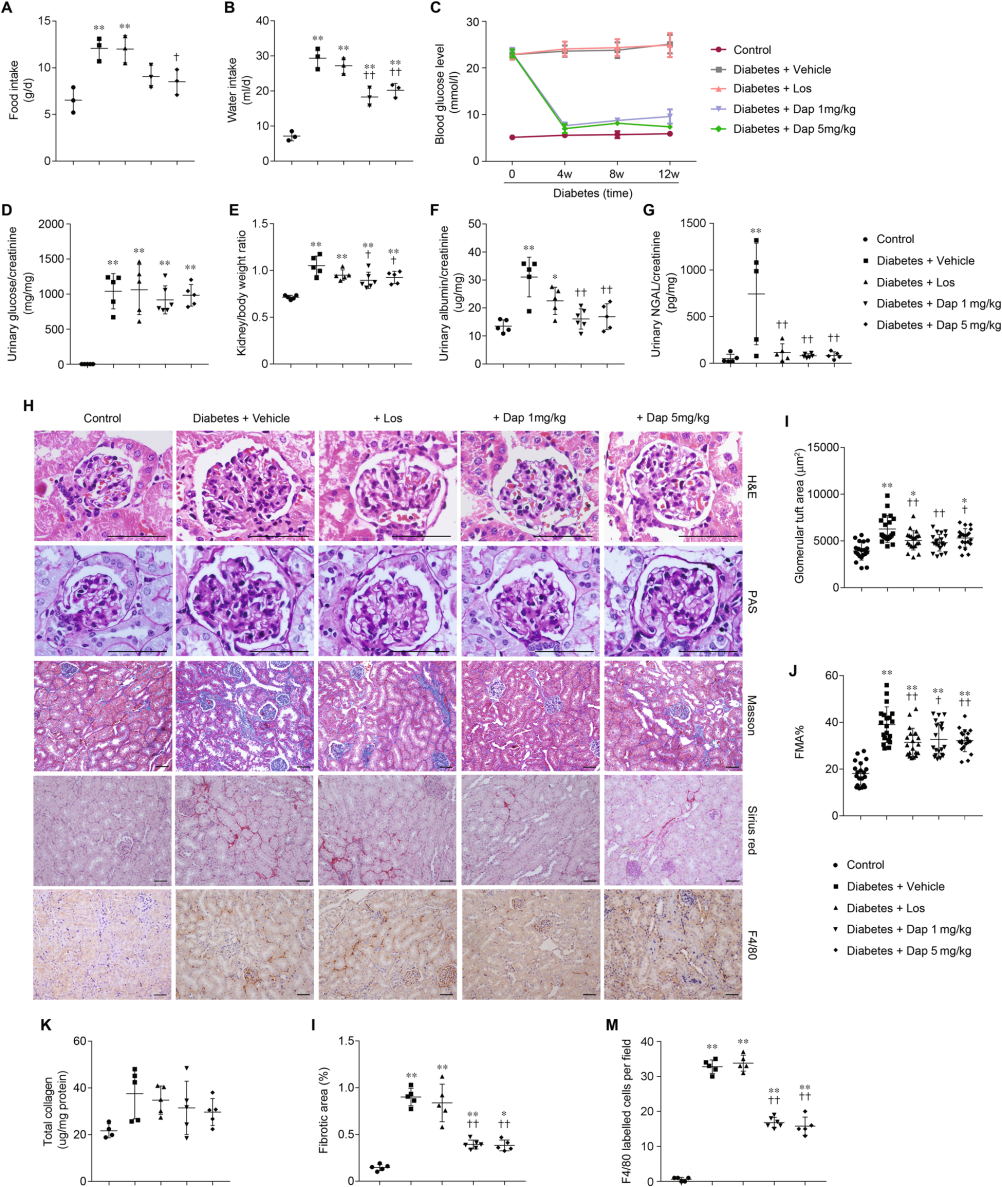
Administration of dapagliflozin ameliorates glomerular and tubular damage of diabetic kidney. **a**, **b** Data of food intake (**a**) and water intake (**b**) at 12 weeks of treatment. **c** Time course data of blood glucose. **d**–**g** Data of urinary glucose (**d**), kidney/body weight ratio (**e**), urinary albumin (**f**), urinary NGAL (**g**) at 12 weeks of treatment. Data are shown as mean ± SD. *p < 0.05 compared with control; **p < 0.01 compared with control; ^†^*p* < 0.05 compared to diabetes + vehicle. ^††^*p* < 0.01 compared with diabetes + vehicle. **h** Representative images of mice kidney sections from the groups indicated with H&E, PAS, Masson, Sirius red staining, and immunostaining for F4/80. Scale bar = 50 μm. **i**–**m** Glomerular tuft area (**i**), fractional mesangial area (FMA) % (**j**), total collagen (**k**), fibrotic area (**l**), and F4/80 lebelled cells per field (**m**) at 12 weeks of treatment. Data are shown as mean ± SD. *p < 0.05 compared with control; **p < 0.01 compared with control; ^†^*p* < 0.05 compared with diabetes + vehicle; ^††^*p* < 0.01 compared with diabetes + vehicle.

We first characterized the phenotypes of each mouse group. Food and water intake in diabetic group was approximately two- and threefold greater than that in the control group, respectively. Dapagliflozin treatment markedly reduced the food (Fig. 5a) and water intake (Fig. 5b) in diabetic group. The blood glucose levels in dapagliflozin groups were significantly lower than that in the vehicle group and losartan group, and comparable with that in the control group at 4, 8, and 12 weeks of treatment (Fig. 5c). Diabetic mice excreted a significant amount of glucose in the urine, whereas control mice did not exhibit such glucosuria (Fig. 5d). By contrast, the urinary glucose levels in dapagliflozin groups were significantly higher than that in the control group, and comparable with that in the vehicle and losartan group at 12 weeks, perhaps reflecting that the effects of reduction in blood glucose levels were eliminated by the inhibition of glucose reabsorption in the dapagliflozin groups. The increase of kidney/body weight ratio in diabetic group was reduced by dapagliflozin treatment (Fig. 5e). Urinary excretion of albumin and NGAL level were remarkably increased in diabetic group, reflecting dysfunction of glomerular filtration barrier and tubular epithelial cells. Both dapagliflozin and losartan treatment significantly reduced the urinary albumin (Fig. 5f) and NGAL (Fig. 5g) levels at 12 weeks. Morphological analysis (Fig. 5h) revealed that glomerular tuft area (Fig. 5i), mesangial expansion, and mesangial matrix hyperplasia (fractional mesangial area, FMA) (Fig. 5j), which were increased in diabetic mice, were also equally improved by dapagliflozin and losartan treatment. However, increased tubulointerstitial fibrosis (Fig. 5k, l) and immune infiltration (Fig. 5m) in diabetic mice were only greatly reduced in dapagliflozin groups, but not in the losartan group. These findings suggest that both dapagliflozin and losartan treatment ameliorate diabetic kidney damage, with a greater tubular protective effect observed in the dapagliflozin group.

### Dapagliflozin protects against metabolic shift from FAO to glycolysis

**Fig. 6:**
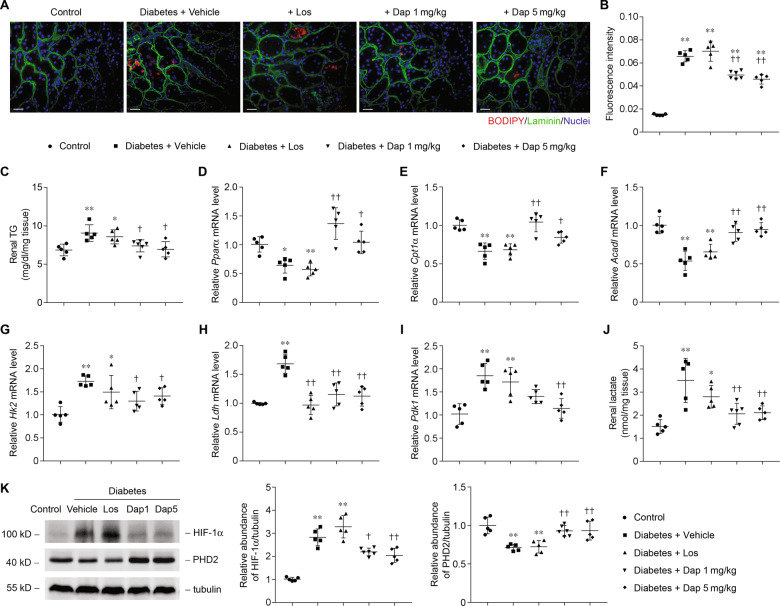
Metabolic switch in diabetic kidneys is attenuated by dapagliflozin treatment. **a** Representative images of mice kidney sections from the groups with BODIPY staining. Scale bars, 25 μm. **b** Quantification of BODIPY positive area in kidney samples from the groups indicated. **p < 0.01 compared with control; ^††^*p* < 0.01 compared with diabetes + vehicle. **c** Triacylglycerol (TG) contents in mouse kidney tissue from the group as indicated. Data are shown as mean ± SD. *p < 0.05 compared with control; **p < 0.01 compared with control; ^†^*p* < 0.05 compared with diabetes + vehicle. **d**–**f** Relative mRNA levels of *Pparα* (**d**), *Cpt1α* (**e**), and *Acadl* (**f**) in samples from the groups indicated. *p < 0.05 compared with control; **p < 0.01 compared with control; ^†^*p* < 0.05 compared with diabetes + vehicle; ^††^*p* < 0.01 compared with diabetes + vehicle. **g**–**i** Relative mRNA levels of *Hk2* (**g**), *Ldh* (**h**), and *Pdk1* (**i**) in samples from the groups indicated. *p < 0.05 compared with control; **p < 0.01 compared with control; ^†^*p* < 0.05 compared with diabetes + vehicle; ^††^*p* < 0.01 compared with diabetes + vehicle. **j** Quantification of lactate in kidney tissue samples from the groups indicated. *p < 0.05 compared with control; **p < 0.01 compared with control; ^††^*p* < 0.01 compared with diabetes + vehicle. **k** Western blot analysis of HIF-1α and PHD2 protein and relative protein expression level in the groups as indicated. **p < 0.01 compared with control; ^†^*p* < 0.05 compared with diabetes + vehicle; ^††^*p* < 0.01 compared with diabetes + vehicle.

We next test whether lowering the glucose level in tubular epithelial cell protects diabetic mice from metabolic shift. Dapagliflozin-treated animals showed improvement in FAO, as shown by BODIPY-stained kidney sections (Fig. 6a, b) and tissue triacylglycerol level (Fig. 6c). Dapagliflozin treatment restored the expression of *Pparα*, *Cpt1α*, and *Acadl* (Fig. 6d–f). Transcript levels of enzymes involved in glucose metabolism (Fig. 6g–i) and the subsequent tissue lactate level (Fig. 6j) were reduced in dapagliflozin groups. Dapagliflozin did not change the transcript level of *Hif-1α* (Supplementary Fig. 1c), but there were the lower expression of HIF-1α and higher expression of PHD2 in dapagliflozin-treated mice compared with the vehicle group (Fig. 6k). No such significant changes were observed between vehicle and losartan group.

**Fig. 7:**
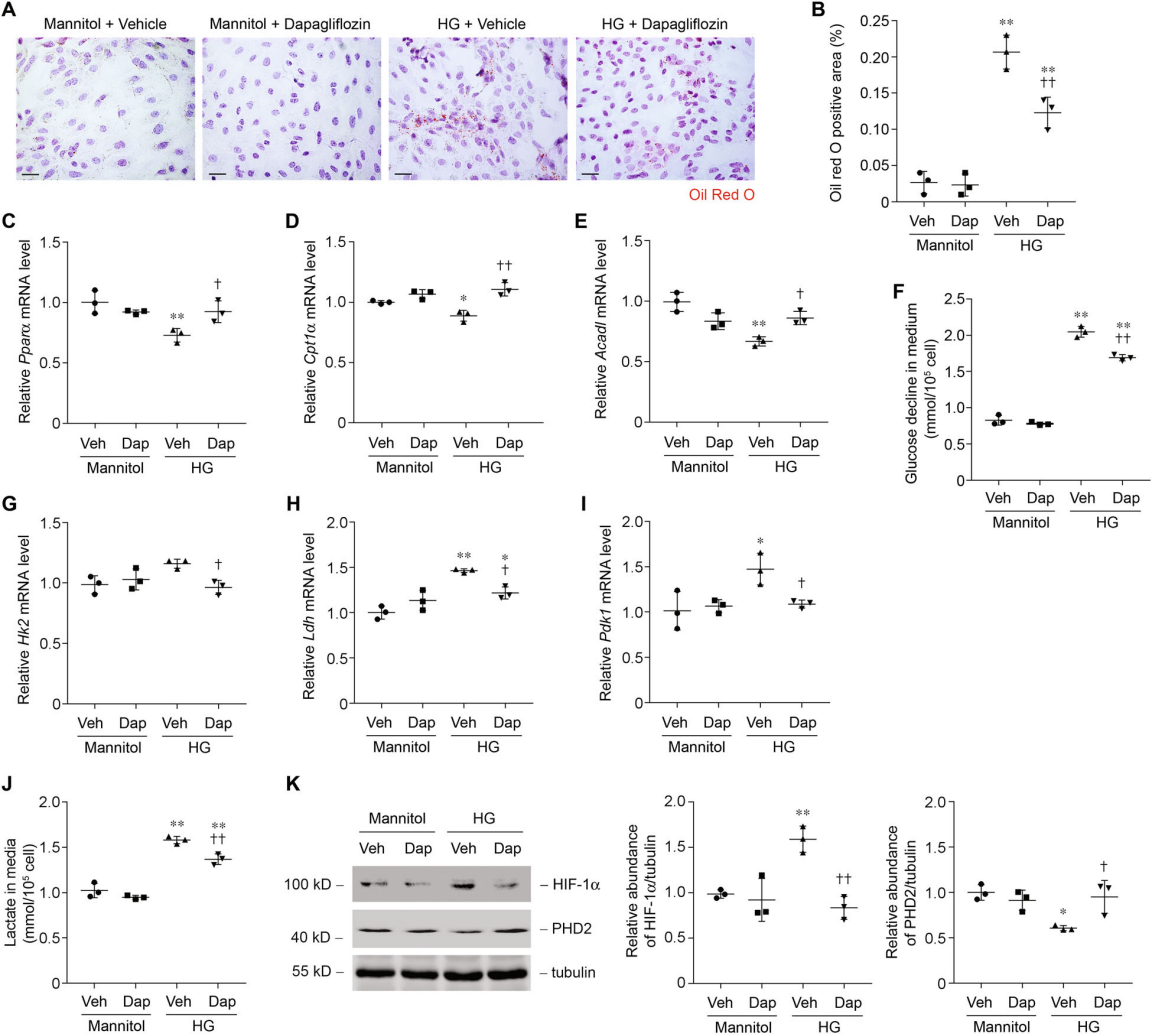
Dapagliflozin treatment prohibits high glucose (HG)-induced metabolic switch in primary tubular epithelial cells (PTCs). **a** Representative images of PTCs incubated with glucose (30 mmol/l) for 24 h in the presence or absence of dapagliflozin (2 μmol/l) with Oil Red O staining. Scale bars, 25 μm. **b** Quantification of Oil Red O-positive area in PTCs incubated with glucose for 24 h in the presence or absence of dapagliflozin. *n* = 3 experiments. **p < 0.01 compared to control; ^††^*p* < 0.01 compared with HG + vehicle. **c**–**e** Relative mRNA levels of *Pparα* (**c**), *Cpt1α* (**d**), and *Acadl* (e) in PTCs incubated with glucose for 6 h in the presence or absence of dapagliflozin. *p < 0.05 compared with control; **p < 0.01 compared with control; ^†^*p* < 0.05 compared with HG + vehicle. ^††^*p* < 0.01 compared with HG + vehicle. **f** Glucose decline in medium of PTCs incubated with glucose for 24 h in the presence or absence of dapagliflozin. **p < 0.01 compared with control; ^††^*p* < 0.01 compared with HG + vehicle. **g**–**i** Relative mRNA levels of *Hk2* (**g**), *Ldh* (**h**), and *Pdk1* (**i**) in PTCs incubated with glucose for 6 h in the presence or absence of dapagliflozin. *p < 0.05 compared with control; **p < 0.01 compared with control; ^†^*p* < 0.05 compared with HG + vehicle. **j** Quantification of lactate in culture medium of PTCs incubated with glucose for 24 h in the presence or absence of dapagliflozin. **p < 0.01 compared with control; ^††^*p* < 0.01 compared with HG + vehicle. **k** Western blot analysis of HIF-1α and PHD2 protein and relative protein expression level in PTCs incubated with glucose for 6 h in the presence or absence of dapagliflozin. *p < 0.05 compared with control; **p < 0.01 compared with control; ^†^*p* < 0.05 compared with HG + vehicle; ^††^*p* < 0.01 compared with HG + vehicle.

We next tested whether HG-induced metabolic shift was reduced by dapagliflozin using cultured PTCs. Dapagliflozin treatment reduced HG-induced lipid accumulation (Fig. 7a, b), normalized *Pparα* repression and FAO enzymes levels reduction (Fig. 7c–e). As expected, glucose decline in medium was inhibited by dapagliflozin (Fig. 7f). Concomitantly, glycolysis enzyme-level elevation (Fig. 7g–i) and lactate production (Fig. 7j) were relieved by dapagliflozin. Dapagliflozin also ameliorated HG-induced expression of HIF-1α and depression of PHD2 (Fig. 7k). These results indicated that dapagliflozin treatment may exhibit renal protection by ameliorating metabolic shift from FAO to glycolysis in proximal tubule cells.

### Dapagliflozin protects against HG-induced metabolic shift in PTCs via inhibiting HIF-1α

**Fig. 8:**
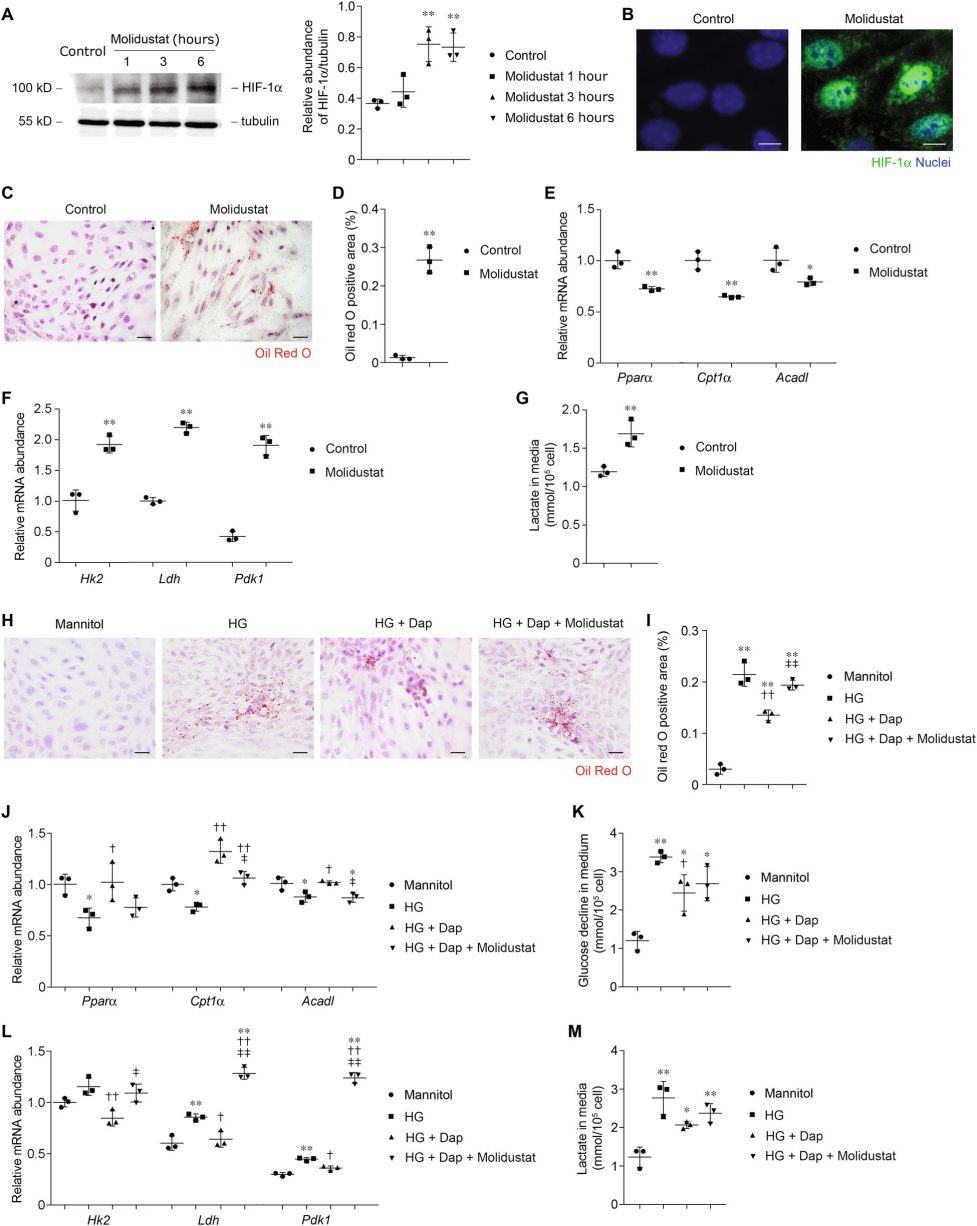
Dapagliflozin protects against metabolic switch by inhibition of HIF-1α. **a** Western blot analysis of HIF-1α protein and relative HIF-1α protein expression level in PTCs incubated with 10 μmol/l of molidustat for various time periods as indicated. *n* = 3 experiments. **p < 0.01 compared with control. **b** Representative images of PTCs incubated with 10 μmol/l of molidustat for 6 h with immunofluorescent staining for HIF-1α. Scale bar = 10 μm. **c** Representative images of PTCs incubated with 10 μmol/l of molidustat for 24 h with Oil Red O staining. Scale bar = 25 μm. **d** Quantification of Oil Red O-positive area in PTCs incubated with 10 μmol/l of molidustat for 24 h. **p < 0.01 compared with control. **e** Relative mRNA levels of *Pparα*, *Cpt1α*, and *Acadl* in PTCs incubated with 10 μmol/l of molidustat for 6 h. *p < 0.05 compared with control; **p < 0.01 compared with control. **f** Relative mRNA levels of *Hk2*, *Ldh*, and *Pdk1* in PTCs incubated with 10 μmol/l of molidustat for 6 h. **p < 0.01 compared to control. **g** Quantification of lactate in the culture medium of PTCs incubated with 10 μmol/l of molidustat for 24 h. **p < 0.01 compared with control. **h** Representative images of PTCs incubated with glucose (30 mmol**/**l) and dapagliflozin (2 μmol/l) in the presence or absence of molidustat (10 μm) for 24 h with Oil Red O staining. Scale bar = 25 μm. **i** Quantification of Oil Red O-positive area in PTCs as indicated. **p < 0.01 compared with control. ^††^*p* < 0.01 compared to HG; ^‡‡^*p* < 0.01 compared with HG + dapagliflozin. **j** Relative mRNA levels of *Pparα*, *Cpt1α*, and *Acadl* in PTCs, as indicated. *p < 0.05 compared with control; ^†^*p* < 0.05 compared with HG. ^††^*p* < 0.01 compared with HG. ^‡^*p* < 0.05 compared with HG + dapagliflozin. ^‡‡^*p* < 0.01 compared with HG + dapagliflozin. **k** Glucose decline in medium of PTCs as indicated. *p < 0.05 compared with control. **p < 0.01 compared with control. ^†^*p* < 0.05 compared with HG. **l** Relative mRNA levels of *Hk2*, *Ldh*, and *Pdk1* in PTCs as indicated. **p < 0.01 compared with control. ^†^*p* < 0.05 compared to HG; ^††^*p* < 0.01 compared with HG; ^‡^*p* < 0.05 compared with HG + dapagliflozin; ^‡‡^*p* < 0.01 compared with HG + dapagliflozin. **m** Quantification of lactate in the culture medium of PTCs incubated with glucose (30 mmol/l) and dapagliflozin (2 μmol/l) in the presence or absence of molidustat (10 μmol/l) for 24 h. *p < 0.05 compared with control; **p < 0.01 compared with control.

The in vivo and in vitro data pointed to a molecular model in which glucose induces a HIF-1α-driven metabolic program via regulation of HIF-1α-target genes. Dapagliflozin prohibited glucose-induced HIF-1α expression and the consequent metabolic shift. To test this hypothesis, we incubated PTCs with the HIF stabilizer molidustat, which was previously shown to inhibit PHD and thereby stabilize HIF-1α in vivo and in vitro^27,28^. In PTCs, expression of HIF-1α was rapidly increased after molidustat treatment (Fig. 8a). Molidustat also induces the nuclei location of increased HIF-1α in PTCs (Fig. 8b). Molidustat treatment was associated with metabolic changes in PTCs as demonstrated by more intracellular lipid-droplet accumulation (Fig. 8c, d). The *Pparα* mRNA levels and those of its downstream targets *Cpt1α* and *Acadl* were consistently reduced by molidustat (Fig. 8e). In contrast, levels of glucose metabolism-related enzymes were higher in molidustat-treated PTCs (Fig. 8f). Level of lactate, a direct measurement of glycolysis, was higher after molidustat treatment (Fig. 8g). These results collectively suggest that molidustat treatment may cause cell metabolic switch, a similar phenotype that is observed in HG-treated cells.

Interestingly, the effects of dapagliflozin on glucose-incubated PTCs, such as reducing lipid accumulation (Fig. 8h, i), reduction of *Pparα* and FAO expression (Fig. 8j) were reversed by molidustat. Despite the fact that the uptake and subsequent utilization of glucose in PTCs were blocked by dapagliflozin (Fig. 8k), levels of glycolysis enzymes (Fig. 8l) and lactate production (Fig. 8m) were still significant increased in the presence of molidustat. This finding suggest a major role for dapagliflozin in metabolic regulation in PTCs, in which dapagliflozin inhibits HIF-1α expression and reprograms metabolic gene profile.

## Discussion

In this study, we have demonstrated that tubular epithelial cells from diabetic patients and mice with experimental model of diabetes undergo a metabolic switch from FAO to glycolysis, and the underlying mechanism for this metabolic switch is likely involved in stabilization of HIF-1α. Notably, this metabolic alteration in the diabetic kidney was ameliorated by SGLT2 inhibition, suggesting that reabsorption of excessive glucose may be the key for the metabolic switch from FAO to glycolysis in renal tubule cells under diabetic condition. Consistent with this, stabilization of HIF-1α in the tubule cells of diabetic kidneys was also nullified by SGLT2 inhibition. Reduced metabolic disorders in the tubule cells by SGLT2 inhibition was accompanied by the alleviation of hyperfiltration, mesangial expansion, albuminuria and tubulointerstitial fibrosis and immune infiltration. Although SGLT2 inhibition lowers blood glucose levels and may contribute to these preferable effects, the probability that SGLT2 inhibitor can directly protect the proximal tubule merits investigation.

SGLT2 is expressed almost exclusively in proximal convoluted tubular epithelial cells and is responsible for reabsorption of more than 90% of filtered glucose. The remaining 10% of escaped glucose is subsequently reabsorbed by SGLT1 expressed in the straight descending proximal tubular epithelial cells^29,30^. Diabetes-induced changes in expression and transporters activity of SGLTs remain controversial; however, the principle role of SGLT2 in glucose reabsorption is well established^30^. The half-maximal inhibitory concentration of dapagliflozin for SGLT2 is less than 1000th of that for SGLT1, so that the drug has high specificity for SGLT2 inhibition and does not interfere with intestinal glucose absorption^31^. The unexpected kidney protection effects of SGLT2 inhibitor was discovered in clinical trials for evaluation their cardiovascular safety^9,32,33^. The explanation of the kidney protective role of SGLT2 inhibition is normalizing glomerular hemodynamics and reversing afferent vasodilation by lowering sodium and chloride reabsorption in proximal tubule and consequently reactivates tubuloglomerular feedback by restoring solute delivery to the macula densa^34^. The hemodynamic effect is consistent with the reduction of albuminuria and risk for doubling of serum creatinine level. In addition to hemodynamic effects, previous studies have indicated that SGLT2 inhibition could decrease glucose uptake through proximal tubule, mitigate-related tubulointerstitial injury, and thereby protect diabetic kidney^35,36^. However, the tubular protective mechanism is not quite understood. In this study, in contrast to glomerular changes which were mitigated by dapagliflozin and losartan treatment, tubulointerstitial lesions, including fibrosis and macrophage infiltration, were reduced only by dapagliflozin, but not by losartan treatment. This suggested that besides hemodynamic effect, SGLT2 inhibition has additional therapeutic effects probably by normalizing metabolic shift to ameliorate tubular injury.

The kidneys have vast amount of mitochondria and consume second-highest level of molecular oxygen at rest^13^. Most of the oxygen is metabolized to produce ATP by mitochondrial oxidative phosphorylation (OXPHOS). In the kidney tubulointerstitial compartment under healthy conditions, the majority of the ATP is generated via fatty acid oxidation. Moreover, the kidney produced ATP is mainly used for power tasks such as reabsorption of glucose, sodium, ions, and other metabolites from filtered urine^37–39^, which are primarily performed by proximal tubule. Other ATP-consuming processes, such as contraction of mesangial cells and podocytes and synthesis of hormone and protein, require substantially lower ATP than those of tubule cells. The synergistic loop between ATP production and utilization is balanced by oxygen, kidney blood flow, and metabolite reabsorption^40,41^. In diabetes, changes of sodium handling in the proximal tubule are major contributor to alteration in GFR^42^, indicating a tubulocentric view of DKD. This view is further supported by the premise that renal tubule injury is a vital determinant of the progression to ESRD^6,43^. Proximal tubule cells depend critically on FAO as their energy source, whereas depressed FAO is associated with higher lipid accumulation in tubule cells from kidneys of individuals and animals with diabetes. Excessive glucose overload in tubule cells causes defective FAO, which has a key role in tubular damage and kidney disease progression as previously reported^44^. Recent study using ob/ob diabetic mice reported that glucose exposure caused metabolic alteration, oxidative stress especially in glomeruli, which were reduced by SGLT2 inhibition. The amelioration of albuminuria, hyperfiltration, and mesangial expansion were also observed in our study; however, tubulointerstitial lesions, including macrophage infiltration, was not ameliorated by ipragliflozin treatment^45^. In their latest study, they mentioned that downregulation of fatty acid metabolism and upregulation glucose metabolism in diabetic renal proximal tubule by HIF-1α stabilizer was protective. Moreover, they suggested that fatty acid metabolisms were upregulated in a very early stage of diabetes^46^. The discrepancy between these reports and our data may be explained by the different animal models and stages of diabetic kidney disease. Nevertheless, in our study, the increased LDH and lactate indicate that glucose is applied mainly for anaerobic glycolysis. The glucose overload-induced metabolic shift from FAO to glycolysis causes tubular dysfunction in diabetes, which makes tubulocentric actually metabolocentric view of DKD^39^.

High-energy demand explains the high oxygen requirement of kidney. Despite the kidneys receive 20% of the cardiac output, kidneys are susceptible to oxygen deprivation. Renal blood and oxygen delivery is regulated by sodium reabsorption^38,41,47,48^. Sodium transport also alters metabolic substrate delivery in the kidney^48–50^. Sodium is reabsorbed along with the glucose in proximal tubule cells, which is also prohibited by SGLT2 inhibition, indicating that SGLT2 inhibition probably affects renal oxygen content. Studies suggested the increased oxygen consumption in diabetic kidney was associated with renal hypoxia^17^. Renal hypoxia is common in individuals with DKD^51^. Our results reveal that SGLT2 inhibition blocks the stabilization of HIF-1α, which may explain its effects on amelioration of metabolic switch in animal DKD model. Luseoglifozin inhibits HIF-1α and subsequent glycolytic genes expression in diabetic kidneys^52^, which is in accordance with the decreased glycolysis enzyme expression and increased lactate from diabetic individuals and animal in our study. However, it was reported that prolyl hydroxylase inhibitor protects against ischemia or obesity-induced kidney injury by modulation of metabolic disorders and inflammation^53,54^. Stabilization of HIF-1α induces transcriptional regulation of diverse series of genes contribute to various pathophysiologic changes, such as metabolic switch, extracellular matrix accumulation, apoptosis prevention, and capillaries protection^21,55–57^, which might lead to diverse effects beyond the scope of this study.

In summary, clinical and research efforts have enabled further understanding of the pathogenesis of DKD and potentially uncovered metabolic switch as critical mediator of DKD. In accordance with previous clinical trials showing that SGLT2 inhibition reduces renal events, our findings reveal that SGLT2 inhibition might cause beneficial metabolic effects and protect the proximal tubule in diabetic kidneys. Further studies might warrant obtaining a comprehensive understanding of the therapeutic effects of SGLT2 inhibition on metabolic kidney diseases.

## Supplementary information

Supplement Tables

Supplement figure S1

Supplement figure legend
