# Supplementary material for: Sodium–glucose cotransporter 2 inhibition suppresses HIF-1α-mediated metabolic switch from lipid oxidation to glycolysis in kidney tubule cells of diabetic mice
Source: Cell Death Dis. 2020 May 22;11(5):390. doi: 10.1038/s41419-020-2544-7 (PMC7242894; doi:10.1038/s41419-020-2544-7)
Supplement: Supplementary file 1 — Supplement Tables [file 41419_2020_2544_MOESM1_ESM.docx]

**Supplement Table S1. Clinical characteristics of the enrolled individuals.**

|  | Non-diabetes | Diabetes | *p*-value |
| --- | --- | --- | --- |
| Age (Years) | 41.63 ± 15.51 | 56.83 ± 12.01 | < 0.05 |
| Male (%) | 50% | 71.7% | 0.222 |
| Duration of diabetes (Years) | − | 10.17 ± 7.085 | − |
| SBP (mmHg) | 136.75 ±3.36 | 142.85 ±2.86 | 0.390 |
| DBP (mmHg) | 91.38 ±4.32 | 82.43 ±1.42 | < 0.05 |
| HbA1c (%) | 5.63 ± 0.34 | 9.14 ± 2.21 | < 0.01 |
| Scr (μmol/L) | 73.86 ± 31.60 | 112.20 ± 75.52 | 0.166 |
| eGFR (ml/min/1.73m^2^) | 101.6 ± 27.89 | 75.2 ± 32.95 | < 0.05 |
| ACR (mg/g) | 190.45 ±25.55 | 777.73 ±215.68 | 0.519 |
| PER (g/24h) | 0.76 ±0.23 | 5.46 ±0.86 | < 0.01 |
| Hb (g/L) | 128.13 ±6.00 | 126.48 ±2.93 | 0.827 |
| TG (mmol/L) | 1.31 ±0.23 | 2.04 ±0.26 | 0.255 |
| TC (mmol/L) | 4.52 ±0.43 | 4.83 ±0.30 | 0.675 |
| HDL (mmol/L) | 1.36 ±0.22 | 1.19 ±0.07 | 0.377 |
| LDL (mmol/L) | 2.84 ±0.28 | 2.89 ±0.22 | 0.924 |
| UA (μmol/L) | 309.63 ±18.75 | 322.15 ±14.59 | 0.727 |

HbA1c: hemoglobin A1c; SBP: systolic blood pressure; DBP: diastolic blood pressure; ACR: urinary albumin creatinine ratio; PER: urinary protein excretion rate; Hb: hemoglobin; TG: serum triglycerides; TC: serum total cholesterol; HDL: high-density lipoprotein; LDL: low-density lipoprotein; UA: serum uric acid.

**Supplement Table S2. The sequences of primer pairs.**

| PPARα | (forward) 5’ -CAA GAA TAC CAA AGT GCG ATC AA-3’  (reverse) 5’-ACT GGT AGT CTG CAA AAC CAA A-3’ |
| --- | --- |
| CPT-1α | (forward) 5’-CTC CGC CTG AGC CAT GAA G-3’  (reverse) 5’-CAC CAG TGA TGA TGC CAT TCT-3’ |
| ACADL | (forward) 5’- TCT TTT CCT CGG AGC ATG ACA-3’  (reverse) 5’-GAC CTC TCT ACT CAC TTC TCC AG-3’ |
| HK2 | (forward) 5’-TGA TCG CCT GCT TAT TCA CGG-3’  (reverse) 5’-AAC CGC CTA GAA ATC TCC AGA-3’ |
| LDHA | (forward) 5’-TGT CTC CAG CAA AGA CTA CTG T-3’  (reverse) 5’-GAC TGT ACT TGA CAA TGT TGG GA-3’ |
| PDK1 | (forward) 5’-GGA CTT CGG GTC AGT GAA TGC-3’  (reverse) 5’-TCC TGA GAA GAT TGT CGG GGA-3’ |
| Hif-1α | (forward) 5’-GAA ATG GCC CAG TGA GAA AA-3’  (reverse) 5’-CTT CCA VGT TGC TGA CTT GA-3’ |
| Actin | (forward) 5’-GGC TGT ATT CCC CTC CAT CG-3’  (reverse) 5’-CCA GTT GGT AAC AAT GCC ATG T-3’ |
