# Supplementary figures and images for: Sodium–glucose cotransporter 2 inhibition suppresses HIF-1α-mediated metabolic switch from lipid oxidation to glycolysis in kidney tubule cells of diabetic mice
Source: Cell Death Dis. 2020 May 22;11(5):390. doi: 10.1038/s41419-020-2544-7 (PMC7242894; doi:10.1038/s41419-020-2544-7)

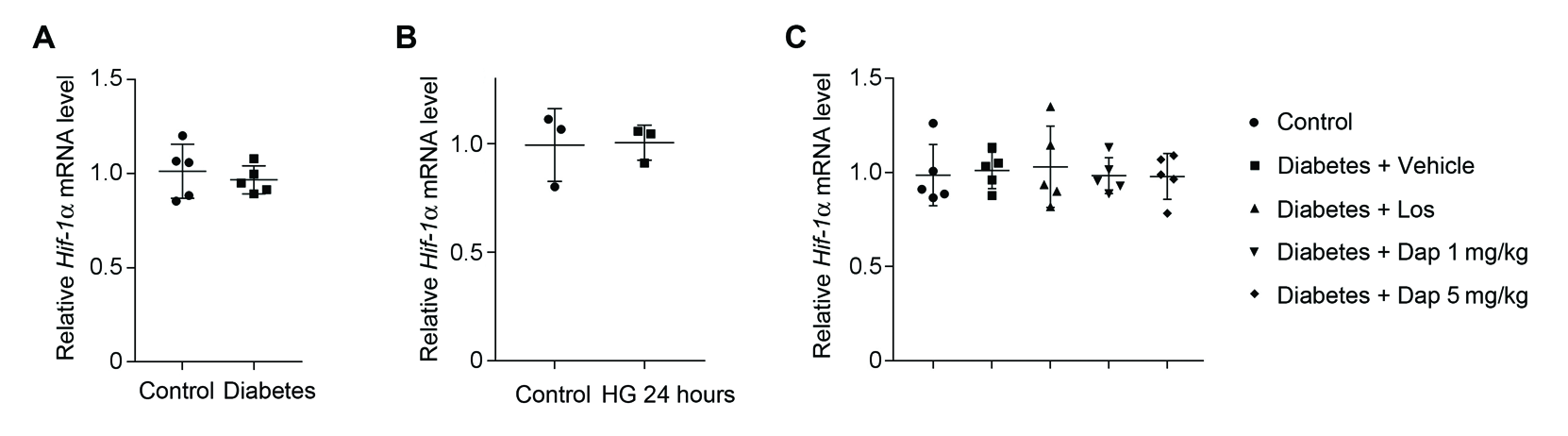

Supplement: Supplementary file 2 — Supplement figure S1 [file 41419_2020_2544_MOESM2_ESM.tif]
