# Supplementary material for: Sodium–glucose cotransporter 2 inhibition suppresses HIF-1α-mediated metabolic switch from lipid oxidation to glycolysis in kidney tubule cells of diabetic mice
Source: Cell Death Dis. 2020 May 22;11(5):390. doi: 10.1038/s41419-020-2544-7 (PMC7242894; doi:10.1038/s41419-020-2544-7)
Supplement: Supplementary file 3 — Supplement figure legend [file 41419_2020_2544_MOESM3_ESM.docx]

**Supplement Figure S1. Q-PCR analysis of HIF-1α in samples of mice kidney and cultured PTCs.** (a) Relative mRNA levels of HIF-1α in control samples and samples from diabetic mice. (b) Relative mRNA levels of HIF-1α in PTCs incubated with 30 mmol/l of glucose for 6 hours. (c) Relative mRNA levels of HIF-1α in the groups indicated.
